# Systematic Analysis of Cinnamyl Alcohol Dehydrogenase Family in Cassava and Validation of *MeCAD13* and *MeCAD28* in Lignin Synthesis and Postharvest Physiological Deterioration

**DOI:** 10.3390/ijms252111668

**Published:** 2024-10-30

**Authors:** Feifei An, Ting Chen, Wenli Zhu, Xinhui Xiao, Jingjing Xue, Xiuqin Luo, Zhuowen Wei, Kaimian Li, Songbi Chen, Jie Cai

**Affiliations:** 1 Tropical Crops Genetic Resources Institute, Chinese Academy of Tropical Agricultural Sciences/Key Laboratory of Ministry of Agriculture for Germplasm Resources Conservation and Utilization of Cassava, Haikou 571101, China; aff85110@163.com (F.A.); zhuwenbamboo@126.com (W.Z.); xiaoxinhui1983@163.com (X.X.); xuetao608@163.com (J.X.); xiuqinluo@163.com (X.L.); zhuowen936@163.com (Z.W.); likaimian@sohu.com (K.L.); 2 National Key Laboratory for Tropical Crop Breeding, Sanya 572025, China; 3 Postgraduate Department, Hainan normal university, Haikou 571158, China; 080107@hainnu.edu.cn

**Keywords:** cassava, cinnamyl alcohol dehydrogenase, gene family, *MeCAD13* and *MeCAD28*, lignin, postharvest physiological deterioration

## Abstract

Cassava (*Manihot esculenta* Crantz) is used as a biomass energy material and an effective supplement for food and feed. Cinnamyl alcohol dehydrogenase (CAD) catalyzes the final step of lignin biosynthesis and is responsible for various stresses. However, systematic investigations of the *CAD* gene family in cassava have been poorly understood. In this study, a genome-wide survey and bioinformatics analysis of *CAD* gene family was performed, transcriptomics, qRT-PCR, gene silencing and stress of yeast cell were used for excavate and validate the candidate *MeCADs* gene. 36 *MeCADs* genes unevenly distributed across 12 chromosomes were identified. Through phylogenetic analyses alongside their *Arabidopsis* counterparts, these MeCADs were divided into four groups, each containing a similar structure and conserved motifs. Interestingly, transcriptome data analysis revealed that 32 *MeCAD* genes were involved in the postharvest physiological deterioration (PPD) process, whereas 27 *MeCAD* genes showed significant changes. Additionally, the relative quantitative analysis of 6 *MeCAD* genes demonstrated that they were sensitive to PPD, suggesting that they may be involved in the regulation of PPD. Silencing *MeCAD13* and *MeCAD28* further showed that lignin content significantly decreased in the leaves. The wound-stress tolerance of transgenic yeast cells was enhanced after transformation with *MeCAD13* and *MeCAD28*. *MeCAD13* and *MeCAD28* may play positive roles in lignin biosynthesis and PPD response, respectively. These results provided a systematic functional analysis of *MeCADs* in cassava and paved a new way to genetically modify lignin biosynthesis and PPD tolerance.

## 1. Introduction

Cassava (*Manihot esculenta* Crantz) is a vital crop that plays a significant role in global food security because of its carbohydrates and essential nutrients, particularly in regions where it serves as a staple food source for millions of people [1]. Its global production was 330 million tons in 2022 (FAOSTAT) [2]. However, cassava faces challenges during postharvest handling and storage, with postharvest physiological deterioration (PPD) being a major concern that affects the quality and market value of tuberous roots [3]. Mechanical damage caused by collision, vibration, and extrusion during harvesting and storage are the primary reason for PPD [4]. Then, a series of biochemical and physiological changes occurs, leading to typical phenomena such as rapid tissue deterioration, discoloration, and loss of firmness. Ultimately, nutritional quality decreases and susceptibility to microbial decay increases [5]. The main research on PPD primarily focuses on reactive oxygen species (ROS) elimination and programmed cell death [6,7,8,9,10]. Furthermore, wound healing can maintain the shelf life of tuberous roots by preventing water loss, nutrient leakage, and pathogen infection [11,12,13]. It has been reported that the wound healing of cassava tuberous roots occurs too late or is insufficient to prevent PPD [14]. Therefore, deepening the research on cassava wound healing has become very necessary.

The phenylpropanoid metabolism pathway regulates wound healing by synthesizing phenolic compounds, lignin, and suberin [15]. For instance, activating the phenylpropane metabolic pathway can promote wound healing in potato tubers [16]. Lignin is a phenolic heteropolymer in secondary cell walls which plays a major role in mechanical support and defense against biotic and abiotic stresses, as well as affecting the quality of fruits [17,18,19,20,21]. In the fields of industry and medicine, lignin is used as an antioxidant for cancer therapies [22] or a coating based on its excellent nanoscale moisture barrier properties [23]. Lignin, with an annual growth of 20 billion tons, offers a huge carbon supply for sustainable industrial methods [24], and the global lignin market will reach USD 6.19 billion as predicted by Radiant Insights [25]. The biosynthesis of lignin involves many enzymes and corresponding genes, in which cinnamyl alcohol dehydrogenase (CAD) catalyzes the final step in monolignol biosynthesis [26]. Decreased lignin content has been reported in CAD down-regulated plants [27,28]. CADs are also involved in various stresses, such as drought and salinity stresses [29,30]. Until now, CAD gene families have been identified in many plants, including Arabidopsis [27], rice [31], poplar [32], mulberry [33], and pear [34]. However, the genome organization, gene structure, and expression profiling in PPD of the *MeCAD* gene family in cassava is poorly understood.

In the present study, we identified the members of the *MeCAD* gene family in cassava, and then analyzed their phylogenetic relationships, gene structures, conserved motifs, synteny, and expression patterns with the PPD transcriptomic data. Additionally, the function of *MeCAD13* and *MeCAD28* in lignin biosynthesis was verified by subjecting transgenic cassava to virus-induced gene silencing (VIGS). The response of *MeCAD13* and *MeCAD28* to wound-stress was also confirmed through yeast stress experiments.

Our findings would identify all the *MeCADs* members at the whole genome level, including their structure and characteristics, and verify the function of *MeCAD* in lignin biosynthesis and PPD. The results can provide a foundation for the in-depth functional analysis of *MeCAD* genes, as well as understanding the mechanisms underlying lignin biosynthesis and PPD regulation. It also provides a theoretical basis for molecular design breeding to improve the lignin content and PPD tolerance of cassava. Accordingly, effective postharvest management strategies can be developed to prolong the shelf life of tuberous roots and minimize PPD losses.

## 2. Results

### 2.1. Identification and Phylogenetic Analysis of CAD in Cassava

A total of 36 CAD proteins were identified from *M. esculenta* and denoted as MeCAD1 to MeCAD36, respectively. All MeCADs contained the CAD domain as confirmed by SMART tests and Pfam analysis (Figure S1). The lengths of MeCAD proteins ranged from 353 (MeCAD28) to 435 (MeCAD17) amino acids, with molecular weights (MW) ranging from 38.41 to 47.36 kDa, and isoelectric points (p*I*) ranging from 5.50 to 8.22. Among the 36 MeCAD proteins, one was predicted to be a peroxisome protein (MeCAD10), five were located in the chloroplast (MeCAD17, 20, 22, 23, and 32), and one was a cytoskeleton protein (MeCAD18). The rest were localized in the cytoplasm. Detailed information on CAD name, gene accession number, chromosome location, protein length, MW, and p*I* for all MeCAD proteins are provided in Table S1.

**Figure 1:**
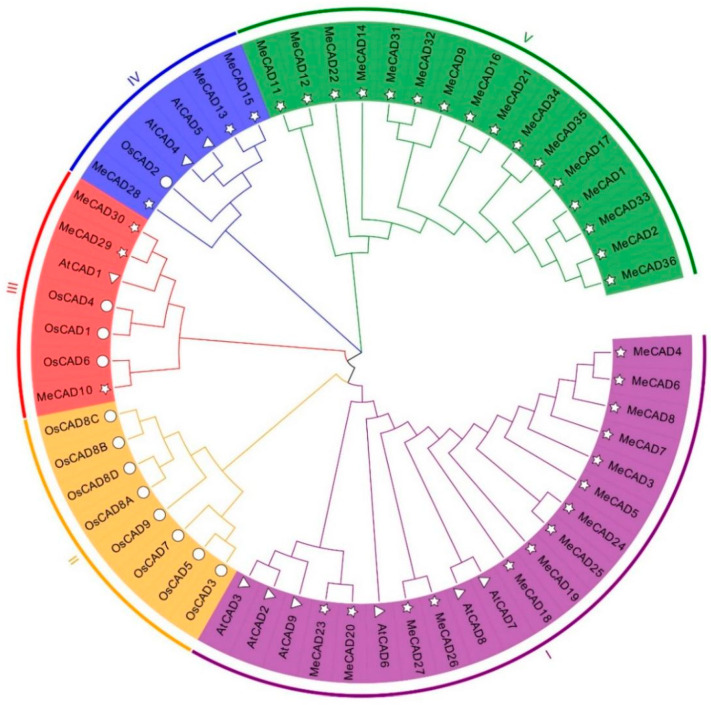
Multiple sequence alignment of CAD domain sequences of *M. esculenta* and *A. thaliana*. ☆ indicate CADs in *M. esculenta*, △ indicate CADs in *A. thaliana.* ○ indicate CADs in *O. sativa.* Group I–V colored with purple, orange, red, blue and green, respectively.

The CAD domain sequences from 36 MeCAD proteins, 12 OsCAD proteins, and 9 AtCAD proteins were utilized to construct a phylogenetic tree using the NJ method. Results indicated that the 36 MeCADs were classified into four groups (Figure 1). Group V contained the highest CAD members with 16 MeCADs, followed by group I with 14 MeCADs. Group IV and Group III had the least CAD members with only 3 MeCADs. However, Group II only includes 8 OsCAD proteins. MeCAD13, MeCAD15, and MeCAD28 were clustered in group IV, including AtCAD4, AtCAD5 and OsCAD2, which were primary genes involved in lignin biosynthesis in *Arabidopsis* [35]. This finding suggested that CAD members in group IV of cassava could be the important genes involved in lignin biosynthesis in cassava.

### 2.2. Chromosomal Locations, Duplications, and Synteny Analysis of the MeCAD Genes

**Figure 2:**
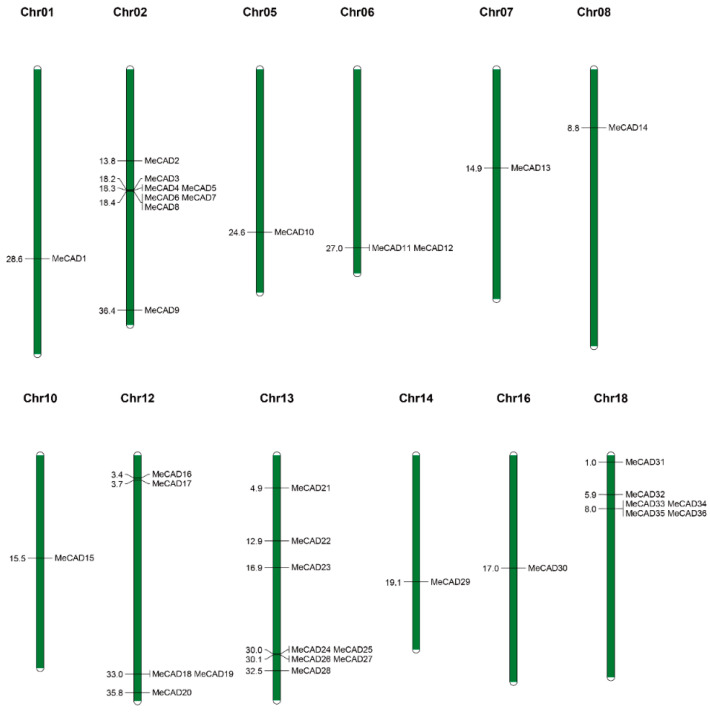
Chromosomal locations of *MeCAD* genes. Green bars represent the *Manihot esculenta* chromosome. The scale bar on the left indicates the lengths of the chromosome (Mb).

**Figure 3:**
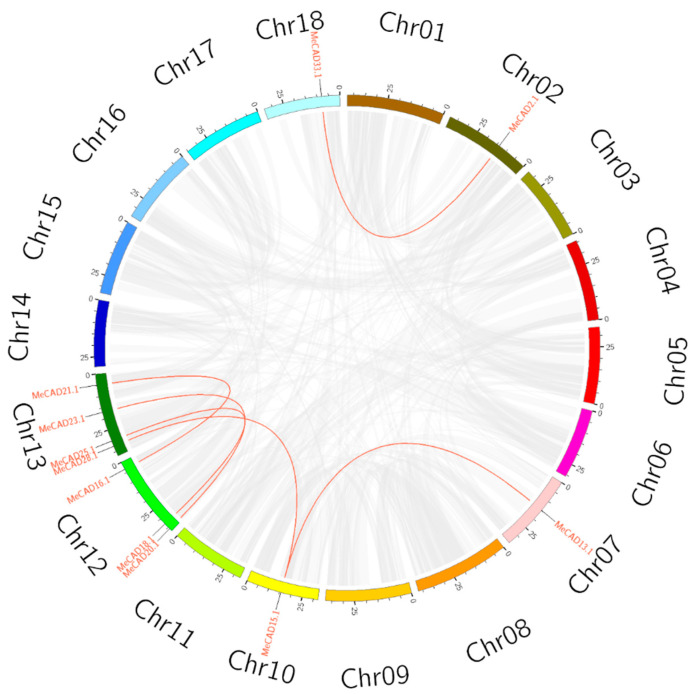
Circos diagram of the *MeCAD* duplication pairs in cassava. The duplication pairs are connected by red lines.

A total of 36 *MeCADs* were mapped on 12 chromosomes, and the distribution on each chromosome was uneven (Figure 2). For instance, chromosomes 02 and 13 contained eight *MeCAD* genes each. However, chromosomes 01, 05, 06, 07, 08, 10, 14, and 16 contained only one *CAD* gene each. Interestingly, many *MeCAD* genes such as those on chromosomes 02, 13, and 18 were clustered in a short distance. Six pairs (*MeCAD2*/*MeCAD33*, *MeCAD13*/*MeCAD15*, *MeCAD15*/*MeCAD28*, *MeCAD16*/*MeCAD21*, *MeCAD18*/*MeCAD25,* and *MeCAD20*/*MeCAD23*) of segmental duplications were identified in total (Figure 3 and Table S2). To detect the collinearity of *CAD* genes, MCScanX was used to analyze the collinearity between *M. esculenta*, *A. thaliana,* and *O. sativa*. It showed 7 pairs of collinearity relationships, involving 5 *MeCAD* and 4 *AtCAD* genes, as well as 1 paired collinearity relationship between 1 *MeCAD* and 1 *OsCAD* (Figure S2 and Table S3).

### 2.3. MeCAD Structure, Conserved Motifs, Cis-Acting Regulatory Element

**Figure 4:**
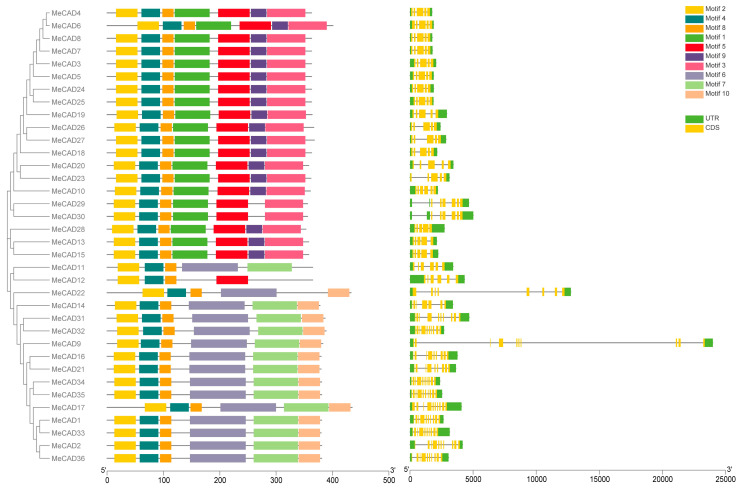
Putative conserved motifs and gene structures of *MeCAD* genes. MEME analysis revealed the conserved motifs of MeCAD proteins. The colored boxes on the right represent 10 motifs. The yellow boxes indicate exons, the black lines represent introns, and the green boxes denote untranslated region (UTR).

The exon/intron architecture and conserved motifs of all *MeCAD* genes were analyzed (Figure 4). The exon numbers ranged from 5 to 10, and 10 *MeCAD* genes contained 10 exons, the highest number of all genes. They included *MeCAD1*, *MeCAD2*, *MeCAD9*, *MeCAD17*, *MeCAD31*, *MeCAD32*, *MeCAD33*, *MeCAD34*, *MeCAD35*, and *MeCAD36.* Meanwhile, *MeCAD14* contained 7 exons, *MeCAD22* contained 8 exons, *MeCAD16* and *MeCAD21* contained 9 exons, *MeCAD10*, *MeCAD11*, *MeCAD12*, *MeCAD29*, and *MeCAD30* contained 6 exons, and the other 17 *MeCAD* genes contained 5 exons. Additionally, *MeCAD* genes in the same group had similar gene structures. Notably, members with high similarity within the same group shared a common motif composition. MeCAD13, MeCAD15, and MeCAD28 were found to contain 7 motifs. Thus, the three genes may have similar functions. MeCAD proteins contain 4–7 conserved motifs. However, only MeCAD12 contained 4 motifs, and 2 MeCAD proteins (MeCAD11, MeCAD22) contained five motifs. A total of 15 MeCAD proteins and 18 MeCAD proteins contain 6 motifs and 7 motifs, respectively.

The *cis*-acting regulatory elements of each *MeCAD* promoter were characterized using PlantCARE and PLACE databases. The 10 most *cis*-acting regulatory elements were found in *36 MeCADs* (Figure S3). All 36 *MeCAD* genes exhibited the common regulatory elements such as CAAT-box and TATA-box. We also identified *cis*-acting regulatory elements in correlation with hormone responses, namely, MYB, ABRE, and ARE, which were involved in abscisic acid (ABA) responsiveness, ERE, and MeJA responsive elements. Interestingly, the promoter of 26 *MeCAD* genes contained elements related to wounding (WUN-motif), as well as other elements related to biotic and abiotic stress, such as drought (MBS) and pathogen defense (W-box). In summary, *MeCAD* genes can be regulated by various hormones and different environments.

### 2.4. Expression Profile of MeCADs and Lignin Changes During PPD Process

**Figure 5:**
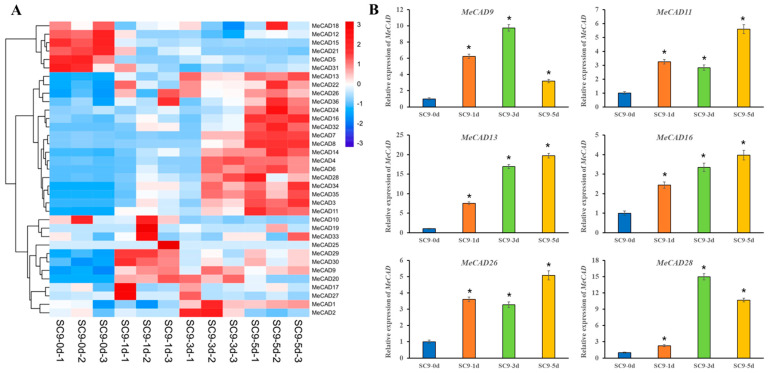
Expression of *MeCAD* genes in tuberous roots during the PPD process and qRT-PCR results for 6 *MeCADs*. (**A**) The heat map was generated by Mev software (V. 4.9.0). The bar on the right indicates relative expression values. The Values 3, 0 and −3 represent high, intermediate, and low expression, respectively. (**B**) qRT-PCR results of 6 up-regulated *MeCAD* genes according to RNA-seq. The relative expression levels are standardized to *MeActin*. These data represent the mean of three biological replicates. The x-axis shows four materials from PPD stage. The y-axis represents relative expression levels of *MeCADs*. All data are presented as the means ± SE of three replications. Error bars show the standard deviation. * Significance at 0.05 probability level.

The expression of *MeCADs* during PPD was analyzed by RNA-seq data. A total of 32 (88.89%) *MeCAD* genes exhibited altered expression levels during PPD (Figure 5A). Additionally, 27 *MeCADs* showed significant differences in compared with the control. Among them, 6 up-regulated genes (*MeCAD9*, *MeCAD11*, *MeCAD13*, *MeCAD16*, *MeCAD26*, *MeCAD28*) were verified by qRT-PCR in PPD samples. Thus, the expression of 6 *MeCAD* genes remarkably increased as PPD progressed (Figure 5B). However, the expression levels of *MeCAD9*, *MeCAD28* at 5 d were less than those at 3 d, but still higher than those at 0 d. These results showed no significant difference compared to RNA-seq, where all the expressions were up-regulated relative to those at 0 d.

Lignin biosynthesis was associated with the PPD process, so we also detected the lignin content in tuberous roots during PPD. Results showed that lignin content significantly increased with PPD degree. The lignin content in tuberous roots was 67.45 μg/mg after harvest, whereas on the 1st, 3rd, and 5th after harvest, the values were 69.22, 70.16, and 72.22 μg/mg, respectively (Table 1).

### 2.5. MeCAD13 and MeCAD28 Were Necessary for Lignin Biosynthesis and Wound Response

**Figure 6:**
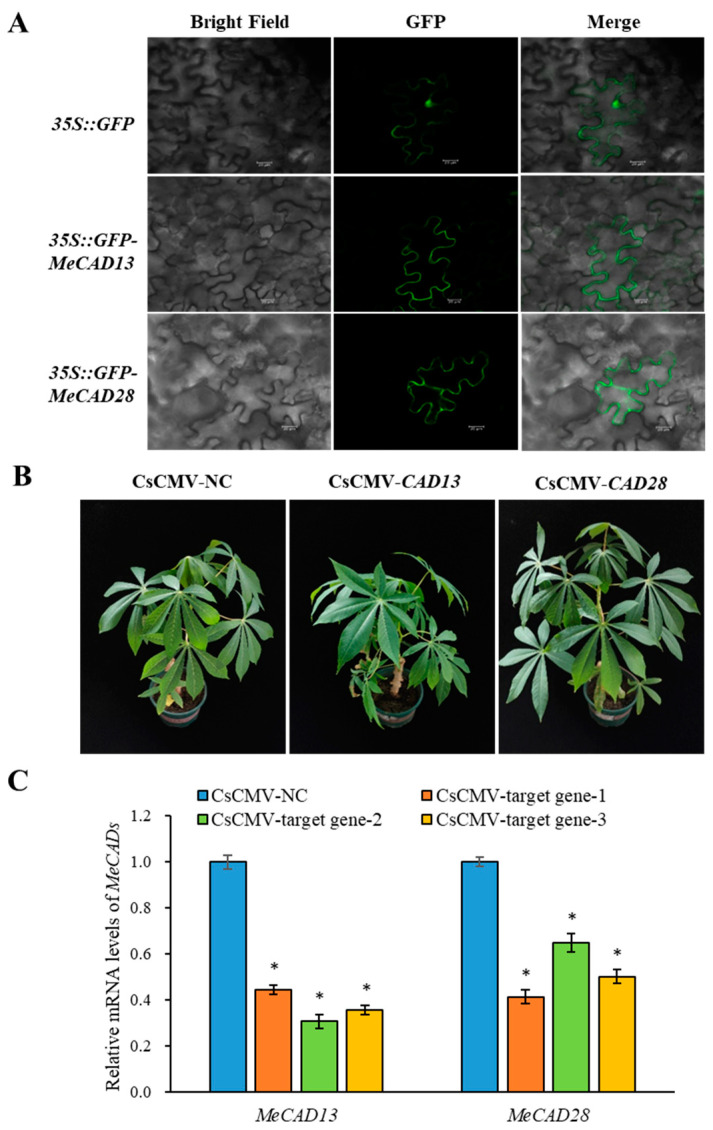
Subcellular localization and silencing of *MeCAD13* and *MeCAD28* using pCsCMV-*CAD13* and pCsCMV-*CAD28.* (**A**) Subcellular localization of MeCAD13 and MeCAD28 in tobacco. Bars = 20 µm. (**B**) Silencing phenotypes using pCsCMV-*CAD13* and pCsCMV-*CAD28* at 25 dpi. CsCMV-NC refers to infected with non-target control pCsCMV-NC. Bars = 1 cm. (**C**) qRT-PCR results of *MeCAD13* and *MeCAD28* mRNA expression infected with pCsCMV-*CAD13* and pCsCMV-*CAD28*. Three independent experiments were performed, with each experiment including three plants per treatment group. Error bars represent the standard deviation. * Significance at 0.05 probability level.

Tissue-specific expression of *MeCAD13* and *MeCAD28* revealed that *MeCAD13* was primarily expressed in tuberous roots and *MeCAD28* was primarily expressed in fibrous roots (Figure S4). We also examined the subcellular localization of MeCAD13 and MeCAD28 proteins in planta. Consistent with previous findings, GFP-fused MeCAD13 protein was localized in the cytoplasm, and GFP-fused MeCAD28 protein was also localized in the cytoplasm in tobacco leaves (Figure 6A), which is consistent with the predictions in Table S1. To investigate the in vivo roles of *MeCADs* in lignin biosynthesis, two gene-silenced cassava lines were generated (*pCsCMV-CAD13* with *MeCAD13* silenced and *pCsCMV-CAD28* with *MeCAD28* silenced). The phenotypes of these *MeCADs*-silenced lines were compared with control plants and shown in Figure 6B. When the transcript levels of *MeCAD13* and *MeCAD28* were assessed in the *MeCAD*-silenced lines, the transcript levels of targeted *MeCADs* were significantly reduced compared to pCsCMV-NC (Figure 6C). The lignin content of *MeCADs*-silenced lines in leaves was compared with that of control plants, and it was found that lignin content significantly decreased in the leaves with *MeCAD13*- and *MeCAD28*-silenced lines (Table 1). In summary, the lignin content in pCsCMV-NC (control) leaves was 25.39 μg/mg, whereas the lignin content in the leaves of *MeCAD28*-silenced lines was 14.89 μg/mg, a decrease of 41.35% compared with the control. However, the lignin content in the leaves of *MeCAD13*-silenced lines decreased more significantly than in *MeCAD28*-silenced lines, reaching 60.06%. Therefore, *MeCAD13* and *MeCAD28* may be candidate genes involved in lignin biosynthesis, and the function of *MeCAD13* in lignin synthesis may be superior to that of *MeCAD28.*

To understand the response of *MeCAD13* and *MeCAD28* to wound stress, the genes were transferred into yeast cells, and the growth of yeast cells was observed by simulating mechanical wound stress with upon systemin addition. As shown in Figure S5, the growth of pDR196-*MeCAD13* and pDR196-*MeCAD28* transgenic yeast strains was better than that of the pDR196 empty vectors after mechanical wound treatment. This indicated that the expression of *MeCAD13* and *MeCAD28* in yeast improved the tolerance of transgenic yeast cells to mechanical wound and enhanced their growth status.

## 3. Discussion

CAD is a major rate-limiting enzyme in lignin biosynthesis. It is related to stress responses and is also closely associated with vegetative tissue and aging [36,37]. The CAD gene family has been identified and functionally analyzed genome-wide in many plant species, such as Arabidopsis [27], rice [31], wheat [38], sweet potato [17], sorghum [39], and tea [40]. The present study aimed to identify and analyze CAD genes in cassava, as well as explore the candidate *MeCADs* genes for regulating lignin synthesis and PPD. A total of 36 *MeCAD* gene members were identified in the cassava genome, and this number was higher than those of Arabidopsis and rice. The reason for the increase in *MeCAD* numbers may be the genome of “AM560” or the gene duplication events.

We subsequently analyzed and predicted gene functions through phylogenetic-tree analysis. Results revealed that 36 *MeCADs* can be classified into four subfamilies (Figure 1), *MeCAD13/15/28* were clustered in cluster IV with *AtCAD4/5* and *OsCAD2*. *AtCAD4* and *AtCAD5* in cluster IV played an important role in the biosynthesis of lignin in *Arabidopsis thaliana* [35], and *OsCAD2* in *Oryza sativa* [41]. We speculated that the *CADs* of cluster IV in cassava may have a function similar to lignin synthesis. In our study, the lignin content significantly decreased in *MeCAD13* silenced and *MeCAD28* silenced leaves, especially in *MeCAD13* silenced lines (Table 1). Therefore, *MeCAD13* and *MeCAD28* were the key genes involved in lignin synthesis in cassava. Furthermore, the homologous *CAD* genes may participate in lignin biosynthesis in one type of tissue during different developmental stages or different tissues [42]. For example, *AtCAD4* was strongly expressed in flowers and roots, and *AtCAD5* was expressed in lignified roots and strongly expressed in pathogen-infected tissues [43]. *MeCAD13* was strongly expressed in tuberous roots, and *MeCAD28* was strongly expressed in fibrous roots (Figure S4). *MeCAD10/29/30* were clustered in cluster III with *OsCAD1/4/6*, consistent with a previous finding that *OsCAD6* is not directly involved in lignin biosynthesis, but may participate in lignans [31]. We speculated that *MeCAD10/29/30* may be correlated with lignans synthesis. No *MeCAD* and *AtCAD* family members were in cluster II with 8 *OsCADs*, and cluster V included only 16 *MeCADs*, which was probably due to the methods and protein sequences. Notably, the function of *MeCADs* in these clusters requires further elucidation.

Gene duplication plays a crucial role in species evolution and gene family evolution [44]. In this study, we identified six pairs of segmental duplications in cassava and no tandem duplications (Figure 3 and Table S2). Tandem duplicated genes were relatively scarce, consistent with the results in tobacco [45]. Thus, segmental duplication was the main duplication model in the cassava genome. A large number of hormone response elements (ABA, MeJA) and stress response elements (wound, drought) were found in *MeCAD* gene promoter (Figure S3). This suggests that *MeCAD* genes may be involved in various stress responses and can be induced by multiple hormones. For instance, ABA has been shown to induce CADs gene expression in sweet potato after exposure to abiotic stress [17]. ABA-induced *CmCADs* expression is reportedly related to the upstream MYB response elements [36], whereas 32 *MeCAD* genes contained MYB elements in promoter regions. Nevertheless, this speculation should be demonstrated by further cloning and function analyses of *MeCAD* promoters. The hormone regulation of different *MeCAD* gene members, and the complex hormonal regulation of *MeCAD* genes under different stress conditions, also warrant further investigation.

The phenylpropanoid pathway genes, including *CADs,* are closely related to biotic and abiotic stresses, including lignin deposition in secondary cell walls and the biosynthesis of defense-related compounds [46,47,48]. As mentioned above, the overexpression of *MeCAD13* and *MeCAD28* also enhanced the wound resistance of yeast (Figure S5). PPD is a kind of mechanical damage caused by a wound. The transcriptional expression and enzyme activity of CAD continuously increased, and a net-like lignin layer formed in wound sites of SC9 tuberous roots [49]. A total of 32 *MeCAD* genes responded to PPD, whereas 27 *MeCAD* genes significantly changed, among which 6 were verified by qRT-PCR (Figure 5). These results were consistent with the changes in CAD enzyme activity in tuberous roots [6]. Lignin accumulation forms a physical barrier to limit pathogen invasion [50], thereby preventing the rapid postharvest deterioration of cassava roots [49]. Lignin content increased in cassava tuberous roots after PPD initiation, and significantly increased with prolonged storage time (Table 1), which may be due to the up-regulation in expression of *MeCAD* genes. Wound healing involves cell wall reinforcement by lignin and other phenolic compounds, in which phenylpropane metabolism plays a vital role [15]. Phenylpropane metabolism was activated after cassava root injury indicated by an increase in phenylalanine ammonia-lyase (PAL) enzymatic activity, coupled with cinnamic acid 4-hydroxylase (C4H), 4-coumarate-CoA ligase (4CL), and CAD [49]. Moreover, MeCAD13 can interact with MePOD12, delaying PPD occurrence through ROS elimination [6]. All these findings indicate that *MeCADs* are closely related with PPD regulation in cassava tuberous roots, whereas the specific functions of *MeCADs* in PPD remain to be demonstrated.

## 4. Materials and Methods

### 4.1. Plant Materials

The tuberous roots of South China 9 (SC9) were harvested 10 months after being planted. They were located in the National Cassava Germplasm Repository, Danzhou, Hainan, China. The tuberous roots were cultivated in a chamber with a 16/8 h photoperiod (day/night) at 26 °C. After 1, 3, and 5 days, the tuberous roots were selected as materials, and PPD score was determined according to Rahmawati et al. [51]; three tuberous roots were used for each time point. All samples were collected and immediately frozen in liquid nitrogen, then stored at −80 °C for further analysis. For VIGS, nine SC9 plants were inoculated with the recombinant VIGS vector 25 days post inoculation (dpi). Leaves, petiole, stem, FEC, flowers, buds, fibrous roots, and tuberous roots of SC9 were used for tissue-specific analysis, and three replicates were used for analysis. *Nicotiana benthamiana* was used for subcellular localization, with three replicates for treatment.

### 4.2. Identification of MeCAD Genes in CASSAVA

The genome sequences (V. 8.1) of cassava were downloaded from the phytozome database (https://phytozome-next.jgi.doe.gov/, accessed on 13 August 2021). The Hidden Markov Model (HMM) profile of CAD (PF00107 and PF08240) was retrieved from Pfam (http://pfam.xfam.org/, V. 37.0). The HMMER program was used for CAD protein search in cassava genome, and then the putative proteins were filtered by e-Value < 0.001 and confirmed by Pfam and SMART database (http://smart.embl-heidelberg.de/, accessed on 10 October 2017). The molecular weight (MW) and theoretical isoelectric point (p*I*) of these proteins were predicted by ExPASy (https://www.expasy.org/, accessed on 1 October 2020). Finally, we named the proteins according to their locations on the chromosomes. Subcellular localization of MeCADs was predicted by WoLF PSORT (https://wolfpsort.hgc.jp/, accessed on 5 January 2007).

### 4.3. Chromosomal Mapping, Gene Structure, Conserved Motif, and Cis-Acting Regulatory Element Analysis

Chromosomal mapping of these *MeCAD* genes was constructed by the MapChart program (V. 2.32). Gene structures were visualized using GSDS2.0 (http://gsds.gao-lab.org/, accessed on 1 April 2015). Additionally, the online software MEME (http://meme-suite.org/tools/meme, V. 5.5.7) was used to identify the conserved motifs among all *MeCAD* genes. The number of motifs was set to 10. Then, 2000 bp upstream sequences of transcription start site ATG were extracted as the promoter sequence and screened using PlantCare (http://bioinformatics.psb.ugent.be/webtools/plantcare/html/, accessed on 1 January 2002) before finally visualizing with GSDS2.0.

### 4.4. Phylogenetic Analysis, Gene Duplication, Multiple Alignments, and Synteny Analysis

Clustal W was performed for the multiple sequence-alignment analysis of CAD domain sequences. MEGA 11 was used to construct the phylogenetic tree with the neighbor-joining (NJ) method. Clustal X (V.2.0) was used to compare the coding sequences of repeated genes. The relationships of duplicated genes were illustrated by Circos. MCScanX was used to detect the *CAD* synteny among *Manihot esculenta*, *Arabidopsis thaliana,* and *Oryza sativa*.

### 4.5. RNA-Seq Analysis and qRT-PCR Verification

The tuberous roots of SC9 were stored for 0, 1, 3, and 5 days and then used for transcriptome analysis. The sequencing platform was Illumina (HiSeq X-Ten, Illumina, Santiago, MN, USA), and three biological replicates were conducted for each sample. Heat maps were created using Mev software (V. 4.9.0). Total RNA was extracted from 3 g of tuberous roots using an RNAprep Pure Plant plus Kit (Tiangen, Beijing, China). One-Step gDNA Removal and cDNA Synthesis SuperMix (TransGen, Shanghai, China) were utilized for first-strand cDNA synthesis. qRT-PCR reactions were carried out in 10 µL volume using a thermocycler (Thermo Fisher Scientific Inc., Göteborg, Sweden). The qRT-PCR primers are listed in Table S4. The reference gene of *MeActin* served as an internal control. Each gene experiment was performed in triplicate for each sample. The relative gene expression levels were calculated using the 2^−ΔΔCt^ method.

### 4.6. Gene Cloning, Expression and Subcellular Localization of MeCAD13 and MeCAD28

Full-length *MeCAD13* and *MeCAD28* were amplified by PCR using the cDNA of SC9 tuberous roots as a template. They were then inserted into the transient expression vector pNC-Green-SubN to produce *35S::GFP-MeCAD13* and *35S::GFP-MeCAD28* recombinant vector, respectively [52]. Table S5 lists the primers used for subcellular localization and sequencing. *Agrobacterium tumefaciens* strain GV3101-psoup was co-transformed with the plasmid, then transiently expressed in *Nicotiana benthamiana* leaves, as described by An et al. [44]. pNC-Green-SubN served as the positive control. After 3 days, the GFP signal (395–509 nm) was observed with a laser scanning confocal microscope (TCS SP8, Leica, Wetzlar, Germany).

### 4.7. Virus-Induced Gene Silencing (VIGS) and qRT-PCR Verification

For vector construction, 300 bp DNA fragments of *MeCAD13* and *MeCAD28* were cloned into pCsCMV-NC, as illustrated by Tuo et al. [53]. The primers are listed in Table S5. The recombinant plasmid was co-transformed into *A. tumefaciens* strain GV3101-psoup and cultured at 28 °C in a growth chamber. The recombinant strain was resuspended in a solution containing 10 mM MES, 10 mM MgCl_2_, and 100 μM acetosyringone. The preparations were infiltrated into the back of cassava leaves using a syringe and grown in greenhouse for 23 days. The expression level of the target gene in silenced lines was subsequently detected, with the qRT-PCR primers shown in Table S4.

### 4.8. Quantitative Analysis of Lignin

The lignin content in tuberous roots and leaves of cassava were measured as described in Foster et al. [54], with minor modification. The main method involved using acetyl bromide to dissolve lignin in the sample, followed by determining the total lignin content through a colorimetric method. A multi-mode microplate reader (Multiskan GO, Thermo Fisher Scientific, Waltham, MA, USA) was used for the quantitative analysis of lignin content.

### 4.9. Stress of Yeast W303

The CDS of *MeCAD13* and *MeCAD28* were amplified (the specific primers are listed in Table S5), and then cloned into the pDR196 vector. The recombinant plasmids of pDR196-*MeCAD13* and pDR196-*MeCAD28* were then transformed into *Saccharomyces cerevisiae* W303. The yeast cells were streaked onto SD-Ura medium and grown overnight in liquid SD-Ura medium. After the adjustment to optical density (OD) _600_ = 1.0, the cells were briefly centrifuged for 30 s, the supernatant was discarded, and 20 nM systemin (wound stress) was added. Serial dilutions (OD_600_ = 0.1, 0.01, 0.001) were performed and plated onto SD-Ura medium. The plates were incubated at 30 °C for 3 days and then photographed.

### 4.10. Statistical Analysis

Excel 2019 and Statistical Product and Service Solution program (V. 20, SPSS Inc., Chicago, IL, USA) were used for all statistical analyses. One-way ANOVA (Tukey) was conducted for comparisons of the expression level and lignin content in cassava leaves.

## 5. Conclusions

A genome-wide analysis of the *MeCAD* gene family was conducted, with a specific emphasis on their response to PPD and lignin synthesis. A total of 36 *MeCAD* genes were defined and characterized, unevenly harbored in 12 chromosomes. Four subfamilies were clustered according to protein sequence. Lignin content in the tuberous root increased during PPD, and 27 *MeCAD* genes may play a crucial role in PPD. Among them, *MeCAD13* and *MeCAD28* enhanced wound stress tolerance of transgenic yeast cells. Moreover, silencing *MeCAD13* and *MeCAD28* significantly decreased the lignin content of cassava leaves. The function of *MeCAD13* and *MeCAD28* in PPD could be verified through genetic transformation in the future, and the mechanism of those 2 genes in lignin biosynthesis and PPD should be elaborated, including the transcription factors and interaction proteins with *MeCAD13* and *MeCAD28*. Overall, it will provide a new insight for cassava engineering programs, including improved PPD tolerance and increased lignin content.

## Figures and Tables

**Table 1:** Lignin content in four PPD samples and in leaves infected with CsCMV-*CAD13* and CsCMV-*CAD28*.

Content (μg/mg)	Tuberous Roots				Leaves		
	SC9-0d	SC9-1d	SC9-3d	SC9-5d	CsCMV-NC	CsCMV-*CAD13*	CsCMV-*CAD28*
Lignin	67.45 ± 0.43 d	69.22 ± 0.20 c	70.16 ± 0.44 b	72.22 ± 0.48 a	25.39 ± 0.47 a	10.14 ± 0.20 c	14.89 ± 0.27 b

One-way ANOVA followed by Tukey’s test was conducted to determine the significance of difference, and different letters in the same row indicated extremely significant difference (*p* < 0.05).

## Data Availability

The complete data sets generated in our study have been submitted in the NCBI Sequence Read Archive database (BioProject PRJNA1143580).

## Supplementary Materials

The following supporting information can be downloaded at: https://www.mdpi.com/article/10.3390/ijms252111668/s1.
