# Supplementary material for: Systematic Analysis of Cinnamyl Alcohol Dehydrogenase Family in Cassava and Validation of MeCAD13 and MeCAD28 in Lignin Synthesis and Postharvest Physiological Deterioration
Source: Int J Mol Sci. 2024 Oct 30;25(21):11668. doi: 10.3390/ijms252111668 (PMC11546318; doi:10.3390/ijms252111668)
Supplement: Supplementary file 1 [file ijms-25-11668-s001.zip › ijms-3255029-supplementary.pdf]

Supplementary Materials

Systematic analysis of cinnamyl alcohol dehydrogenase family in cassava and validation of MeCAD13 and MeCAD28 in lignin synthesis and postharvest physiological deterioration

Feifei An[1,2ζ], Ting Chen[3ζ], Wenli Zhu[1], Xinhui Xiao[1], Jingjing Xue[1,2], Xiuqin Luo[1,2], Zhuowen Wei[1], Kaimian Li[1], Songbi Chen[1,2*] , Jie Cai[1,2*]

1. Tropical Crops Genetic Resources Institute, Chinese Academy of Tropical Agricultural Sciences/Key Laboratory of Ministry of Agriculture for Germplasm Resources Conservation and Utilization of Cassava, Haikou 571101, Hainan, China

2. National Key Laboratory for Tropical Crop Breeding, Sanya 572025, Hainan, China

3. Postgraduate Department, Hainan normal university, Haikou 571158, Hainan, China

---

[ζ] These authors contributed equally to this work.

Correspondence: Prof. Songbi Chen, Tropical Crops Genetic Resources Institute, Chinese Academy of Tropical Agricultural Sciences, Haikou, China
Tel: +86-15120690896
Email: songbichen@catas.cn

Correspondence: Associated Prof. Jie Cai, Tropical Crops Genetic Resources Institute, Chinese Academy of Tropical Agricultural Sciences, Haikou, China
Tel: +86-15002029095
Email: caijie@catas.cn

Supplementary Figure S1. Analysis of the conserved domain regions in the 36 MeCADs.

Supplementary Figure S2. Synteny analysis of *CAD* genes between *M. esculenta, O. sativa* and *A. thaliana.* Gray lines in the background represent the collinear blocks within the genomes, while the red lines show the collinear *CAD* gene pairs.

Supplementary Figure S3. Distribution of ten cis-acting regulatory elements in promoter sequences of 36 *MeCAD* genes.

Supplementary Figure S4. Tissue-specific expression of *MeCAD13* and *MeCAD28*. (A) qRT-PCR analyses of *MeCAD13* mRNA expression in leaves, petiole, stem, FEC, buds, fibrous roots and tuberous roots of SC9. (B) qRT-PCR analyses of *MeCAD28* mRNA expression in leaves, petiole, stem, FEC, buds, fibrous roots and tuberous roots of SC9.

Supplementary Figure S5. Wound resistance of transgenic *MeCAD13* and *MeCAD28* yeast cells W303. (A) Cells treated with 20 nM system for 24 h of transgenic *MeCAD13* yeast cells W303. (B) Cells treated with 20 nM system for 24 h of transgenic *MeCAD28* yeast cells W303. pDR196, empty vector; pDR196-CAD13, pDR196-CAD28, transgenic line. Images were captured after incubation at 30 °C for 3 days.

Supplementary Table S1. Primers used for qRT-PCR.

Supplementary Table S2. Specific primers used for CDS amplification and vector construction.

Supplementary Table S3. Detailed information of 36 MeCAD proteins in cassava.

Supplementary Table S4. Pairs of segmental duplications identified in *MeCAD* gene family.

Supplementary Table S5. Paired collinearity relationships between MeCAD, OsCAD and AtCAD.

Supplementary Figure S1. Analysis of the conserved domain regions in the 36 MeCADs.

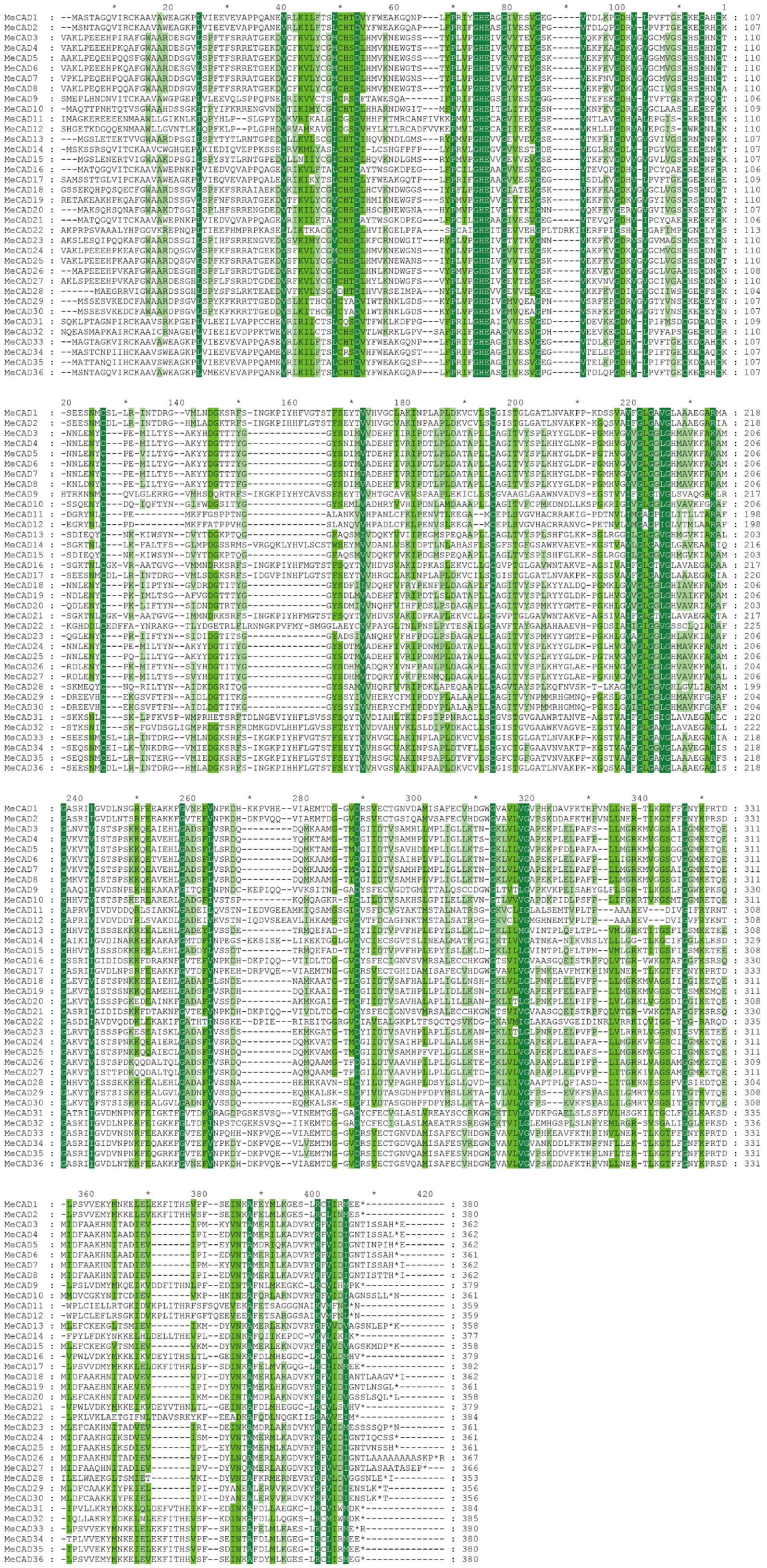

Supplementary Figure S2. Synteny analysis of *CAD* genes between *M. esculenta, O. sativa* and *A. thaliana.* Gray lines in the background represent the collinear blocks within the genomes, while the red lines show the collinear *CAD* gene pairs.

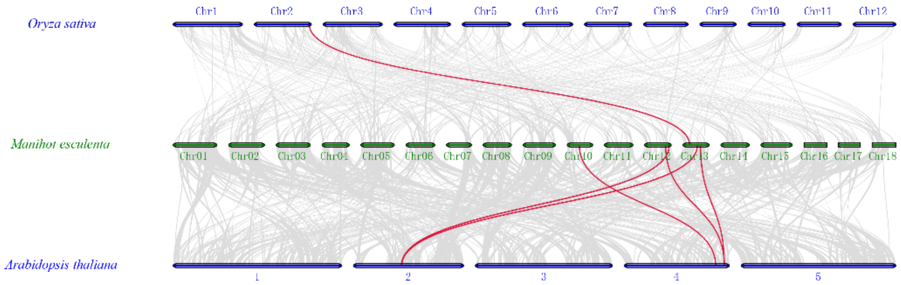

Supplementary Figure S3. Distribution of ten cis-acting regulatory elements in promoter sequences of 36 *MeCAD* genes.

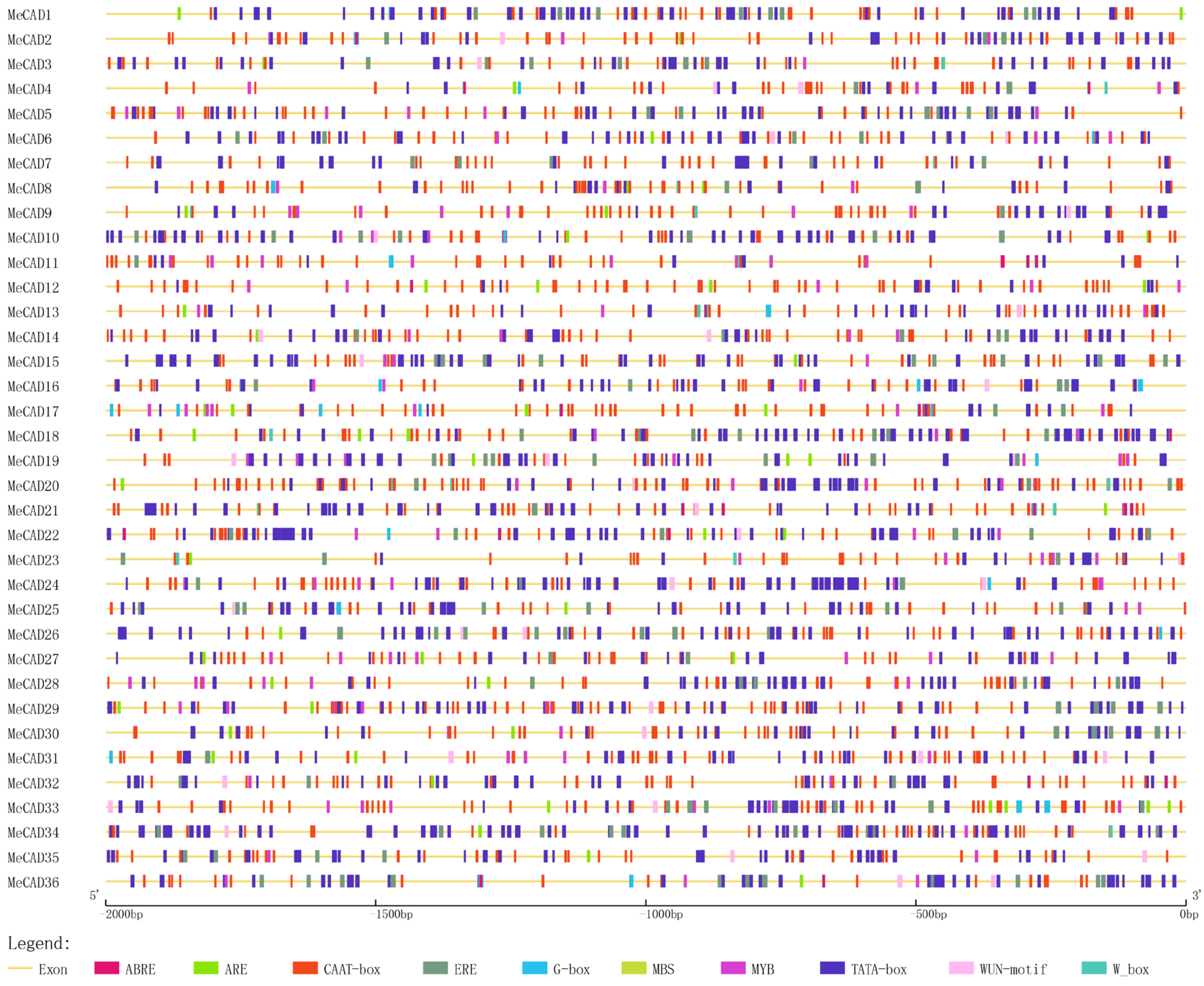

Supplementary Figure S4. Tissue-specific expression of *MeCAD13 and MeCAD28*. (A) qRT-PCR analyses of *MeCAD13* mRNA expression in leaves, petiole, stem, FEC, buds, fibrous roots and tuberous roots of SC9. (B) qRT-PCR analyses of *MeCAD28* mRNA expression in leaves, petiole, stem, FEC, buds, fibrous roots and tuberous roots of SC9.

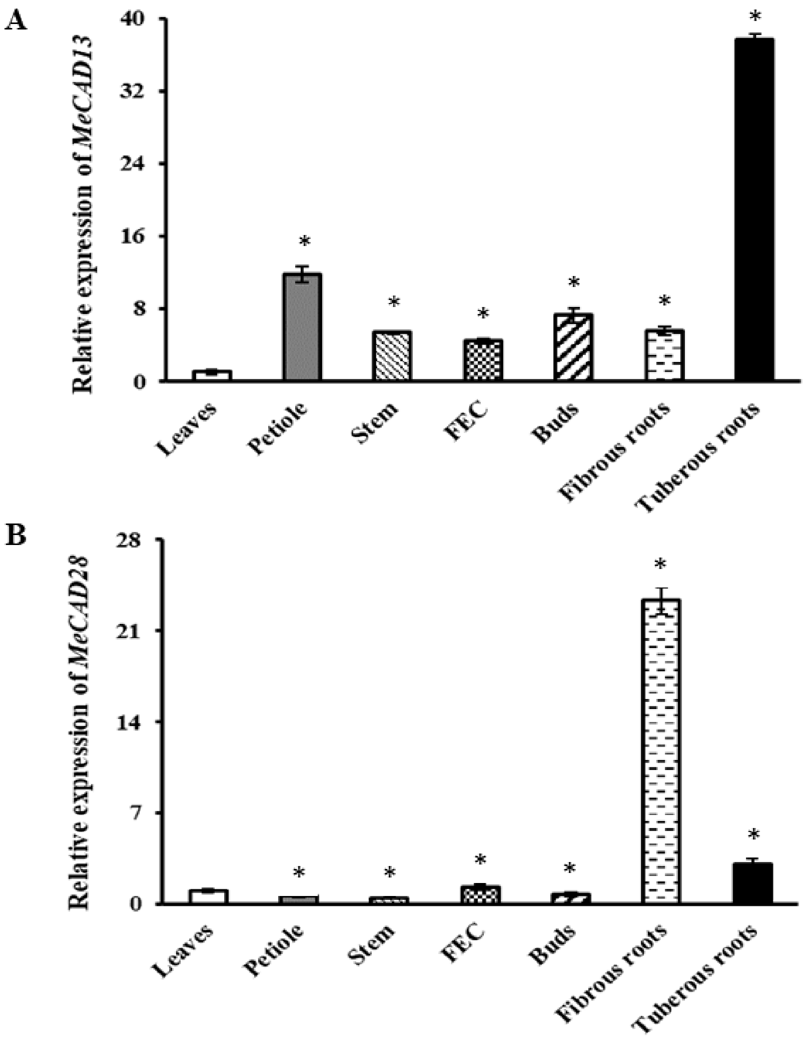

Supplementary Figure S5. Wound resistance of transgenic *MeCAD13* and *MeCAD28* yeast cells W303. (A) Cells treated with 20 nM system for 24 h of transgenic *MeCAD13* yeast cells W303. (B) Cells treated with 20 nM system for 24 h of transgenic *MeCAD28* yeast cells W303. pDR196, empty vector; pDR196-CAD13, pDR196-CAD28, transgenic line. Images were captured after incubation at 30 °C for 3 days.

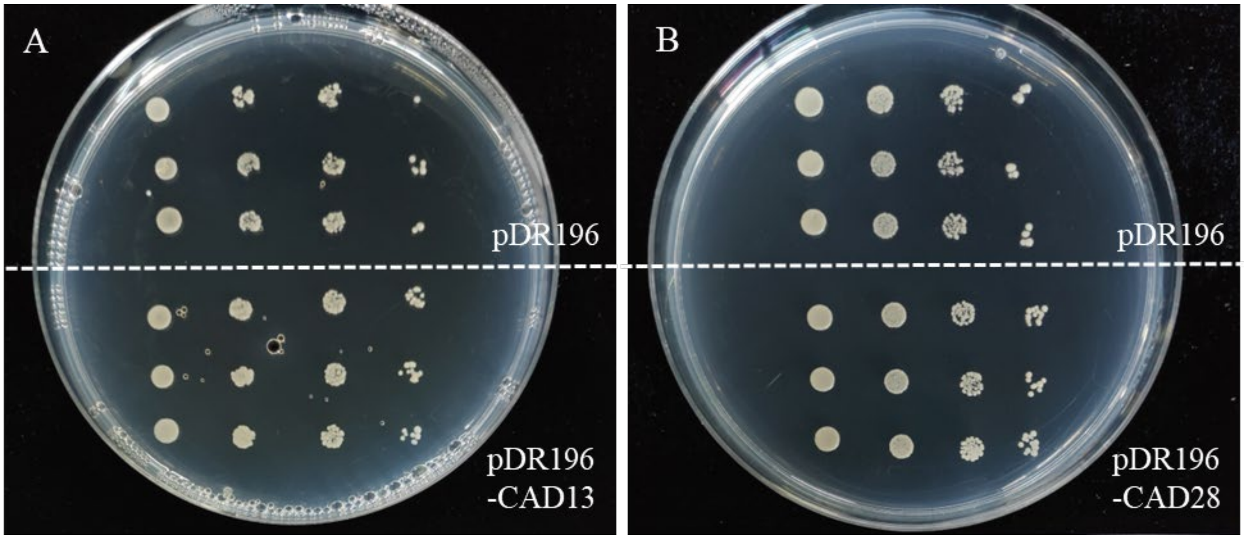

Supplementary Table S1. Detailed information of 36 MeCAD proteins in cassava.

CAD name	gene accession	chromosome locus	length	MV (Da)	pI	subcellular location
MeCAD1	Manes.01G083600	Chromosome01	381	41241.2	6.50	cyto
MeCAD2	Manes.02G174800	Chromosome02	381	41161.1	6.78	cyto
MeCAD3	Manes.02G206300	Chromosome02	363	39274.5	6.87	cyto
MeCAD4	Manes.02G206400	Chromosome02	363	39116.3	6.69	cyto
MeCAD5	Manes.02G206500	Chromosome02	363	39083.9	6.62	cyto
MeCAD6	Manes.02G206600	Chromosome02	401	43786.7	6.96	cyto
MeCAD7	Manes.02G206700	Chromosome02	363	39209.3	6.87	cyto
MeCAD8	Manes.02G206800	Chromosome02	363	39032.1	6.57	cyto
MeCAD9	Manes.02G226001	Chromosome02	383	41303.3	7.02	cyto
MeCAD10	Manes.05G144200	Chromosome05	361	39271	8.22	pero
MeCAD11	Manes.06G142300	Chromosome06	365	39139.2	6.90	cyto
MeCAD12	Manes.06G142400	Chromosome06	365	39181.3	6.43	cyto
MeCAD13	Manes.07G069780	Chromosome07	358	39138.1	5.64	cyto
MeCAD14	Manes.08G063900	Chromosome08	378	40509.6	6.11	cyto
MeCAD15	Manes.10G076700	Chromosome10	358	39108	6.05	cyto
MeCAD16	Manes.12G039100	Chromosome12	380	40774.8	7.18	cyto
MeCAD17	Manes.12G043100	Chromosome12	435	47361.4	7.68	chlo
MeCAD18	Manes.12G122600	Chromosome12	363	38927.7	6.5	cysk
MeCAD19	Manes.12G124000	Chromosome12	364	39238	6.64	cyto
MeCAD20	Manes.12G149800	Chromosome12	358	38543.1	6.93	chlo
MeCAD21	Manes.13G038500	Chromosome13	380	40638.7	7.39	cyto
MeCAD22	Manes.13G076700	Chromosome13	433	46418	7.9	chlo
MeCAD23	Manes.13G081900	Chromosome13	362	38966.6	7.73	chlo
MeCAD24	Manes.13G102200	Chromosome13	363	39203.3	6.88	cyto
MeCAD25	Manes.13G102300	Chromosome13	363	39196.3	6.97	cyto
MeCAD26	Manes.13G102400	Chromosome13	367	39144.8	6.57	cyto
MeCAD27	Manes.13G102500	Chromosome13	368	39471.1	6.08	cyto
MeCAD28	Manes.13G117800	Chromosome13	353	38406.9	6.44	cyto
MeCAD29	Manes.14G152700	Chromosome14	356	38795.3	6.40	cyto
MeCAD30	Manes.16G057118	Chromosome16	356	38795.3	6.40	cyto
MeCAD31	Manes.18G007380	Chromosome18	387	41766	6.59	cyto
MeCAD32	Manes.18G064740	Chromosome18	389	41826	6.32	chlo
MeCAD33	Manes.18G086800	Chromosome18	380	41014.1	6.92	cyto

MeCAD34	Manes.18G086900	Chromosome18	381	41395.4	5.50	cyto
MeCAD35	Manes.18G087000	Chromosome18	381	41403.4	6.01	cyto
MeCAD36	Manes.18G087401	Chromosome18	381	41180	6.69	cyto

Supplementary Table S2. Pairs of segmental duplications identified in *MeCAD* gene family.

Number	Collinear pairs
1	Manes.07G069780: Manes.10G076700
2	Manes.10G076700: Manes.13G117800
3	Manes.12G149800: Manes.13G081900
4	Manes.12G122600: Manes.13G102300
5	Manes.12G039100: Manes.13G038500
6	Manes.02G174800: Manes.18G086800

Supplementary Table S3. Paired collinearity relationships between MeCAD, OsCAD and AtCAD.

Number	Collinear pairs
1	Manes.10G076700: AT4G34230
2	Manes.12G149800: AT2G21730
3	Manes.12G149800: AT2G21890
4	Manes.12G124000: AT4G37970
5	Manes.13G081900: AT2G21730
6	Manes.13G081900: AT2G21890
7	Manes.13G102500: AT4G37970
8	Manes.13G037500: Os02g57250

Supplementary Table S4. Primers used for qRT-PCR.

**Name**	**Forward Primer (5' to 3')**	**Reverse Primer (5' to 3')**
MeActin	TGATGAGTCTGGTCCATCCA	CCTCCTACGACCCAATCTCA
qRT-MeCAD9	TGATTACAACAGCATTACAG	GTCCATAGTGAGCAGATAT
qRT-MeCAD11	AAGAGAAGAAGAAGAGAACAT	CAAGAGAAGGAAGGTGATAA
qRT-MeCAD13	GTTGGAGTTGGAGTTATTG	CATCGGTGTAGACATCAT
qRT-MeCAD16	TTGGCATAGATATTGACAGTA	AAGCAGACCTCATTACAG
qRT-MeCAD26	TCATTCCTGCGACAACTG	AGTAGCCTCCGTAAGTGAT
qRT-MeCAD28	CGGAAGCATAGAAGATAC	AGCCTCATTCACATAATC

Supplementary Table S5. Specific primers used for CDS amplification and vector construction.

Name	Forward Primer (5' to 3')	Reverse Primer (5' to 3')	Notes
MeCAD13	ATGGGTAGCCTTGAAACAGA	TCATGGTTCCAGGTTGCTGC	amplification
MeCAD28	ATGGCGGAAGGAAGAAGGGT	TCATTCGAGGTTGCTTCCAC	amplification
MeCAD13-SubN	agtggtctctgtccagtcct	ggtctcagcagaccacaagt	vector
	ATGGGTAGCCTTGAAACAGA	TCATGGTTCCAGGTTGCTGC	construction
MeCAD28- SubN	agtggtctctgtccagtcct	ggtctcagcagaccacaagt	vector
	ATGGCGGAAGGAAGAAGGGT	TCATTCGAGGTTGCTTCCAC	construction
MeCAD13-VIGS	agtggtctctgtccagtcct	ggtctcagcagaccacaagt	vector
	GAGGTGGGATCAGAGGTCAC	CCCAAAGTGGCTAAGTGGGC	construction
MeCAD28-VIGS	agtggtctctgtccagtcct	ggtctcagcagaccacaagt	vector
	ATGGCGGAAGGAAGAAGGGT	GCATTCCCCACAAGACCAAA	construction
MeCAD13- pDR196	atataccccagcctcg	cgataagcttgatatc	vector
	ATGGGTAGCCTTGAAACAGA	TGGTTCCAGGTTGCTGCCTG	construction
MeCAD28- pDR196	atataccccagcctcg	cgataagcttgatatc	vector
	ATGGCGGAAGGAAGAAGGGT	TTCGAGGTTGCTTCCACCCA	construction
CsCMV	TGGGCGCTAATTAGTTTACTGCA	GGTCAAGACGGCTCAACTCTTCA	sequencing
SubN	CATGGTCCTGCTGGAGTTCGTG	ACCGGCAACAGGATTCAATC	sequencing
pDR196	TTTCTATCAACCTCGTTG	CTTTTCCATCTTTTCGTA	sequencing
